# En Bloc Arch Reconstruction With the Frozen Elephant Trunk Technique for Acute Type a Aortic Dissection

**DOI:** 10.3389/fcvm.2021.727125

**Published:** 2021-09-28

**Authors:** Penghong Liu, Bing Wen, Chao Liu, Huashan Xu, Guochang Zhao, Fuqiang Sun, Hang Zhang, Xingxing Yao

**Affiliations:** Department of Cardiovascular Surgery, The First Affiliated Hospital of Zhengzhou University, Zhengzhou, China

**Keywords:** acute type a aortic dissection, en bloc technique, total arch replacement, frozen elephant trunk, open aortic arch repair

## Abstract

**Objective:** The study objective was to evaluate the effect of en bloc arch reconstruction with frozen elephant trunk (FET) technique for acute type A aortic dissection.

**Methods:** 41 patients with acute Stanford type A dissection underwent en bloc arch reconstruction combined with FET implantation between April 2018 and August 2020. The mean age of the patients was 46 ± 13 years, and 9 patients were female. One patient had Marfan syndrome. Six patients had pericardial tamponade, 9 had pleural effusion, 5 had transient cerebral ischemic attack, and 3 had chronic kidney disease.

**Results:** The hospital mortality rate was 9.8% (4 patients). 2 (4.9%) patients had stroke, 23 (56.1%) had acute kidney injury, and 5 (12.2%) had renal failure requiring hemodialysis. During follow-up, the rate of complete false lumen thrombosis was 91.6% (33/36) around the FET, 69.4% (25/36) at the diaphragmatic level, and 27.8% (10/36) at the superior mesenteric artery level. The true lumen diameter at the same three levels of the descending aorta increased significantly while the false lumen diameter reduced at the two levels: pulmonary bifurcation and the diaphragm. The 1-, 2-and 3-year actuarial survival rates were 90.2% [95% confidence interval (CI), 81.2–99.2], 84.2% (95% CI, 70.1–98.3) and 70.2% (95% CI, 42.2–98), respectively.

**Conclusions:** In patients with acute type A dissection, en bloc arch reconstruction with FET technique appeared to be feasible and effective with early clinical follow-up results. Future studies including a large sample size and long-term follow-up are required to evaluate the efficacy.

## Introduction

Acute type A aortic dissection is a life-threatening cardiovascular disease which remains a challenging procedure in cardiac surgery. Traditional open replacement of the aortic arch requires deep hypothermia circulatory arrest and replacement of the supra-aortic vessels with special consideration for cerebral protection (1). The frozen elephant trunk (FET) technique, combining conventional open surgery with endovascular repair, has been played an important role in treatment of such extensive aortic pathologies (2). Several approaches to arch replacement, such as the separate grafts, hybrid, en bloc (island), and branched stent graft techniques have been applied (3–5). Several reports showed the safety and feasibility of the FET technique with en bloc arch reconstruction for aortic arch disease (5–8). However, a limited number of case studies have reported the treatment of acute aortic A dissection. The aim of this study is to review our experience with the en bloc arch reconstruction and the FET technique for acute type A dissection.

## Materials and Methods

### Patients

From April 2018 to August 2020, en bloc arch reconstruction with FET implantation was performed on 41 consecutive patients with acute Stanford type A dissection involving the descending aorta. All diagnoses were confirmed by echocardiography, computed tomography angiography (CTA), and/or magnetic resonance imaging.

The mean age of the 32 (78%) men and 9 (22%) women was 46 ± 13 years (range, 14–69 years). Six (16.4%) patients had pericardial tamponade and 9 (22%) had pleural effusion. Transient cerebral ischemic attack was present in 5 (12.2%) patients, chronic kidney disease in 3 (7.3%), renal failure requiring hemodialysis in 1 (2.4%), lower extremity ischemia in 2 (4.9%), severe aortic regurgitation in 19 (46.3%), myocardial ischemia in 6 (14.6%) patients and classic Marfan syndrome in one patient. 33 (80.5%) patients had hypertension without effective control. The primary tear site was located in the ascending aorta, aortic arch and proximal descending thoracic aorta in 30 patients, 5 patients and 4 patients, respectively, and an entry tear was not detected in 2 patients. The details of the preoperative patient characteristics are listed in Table 1. This study was performed in compliance with the principles of the Declaration of Helsinki and approved by the Ethics Committee of the First Affiliated Hospital of Zhengzhou University (2020-KY-0310-002), and informed consent from the patients was not needed due to the retrospective nature of this study.

**Table 1:** Preoperative and presentation characteristics.

**Variables**	**All patients** **(***n*** = 41)**
Male gender	32 (78%)
**Age (years)**	
Mean ± SD	46 ± 13
Median (range)	46 (14–69)
**BMI**	
Mean ± SD	26.9 ± 3.1
Median (range)	26.6 (22.0–35.5)
Chest, back or abdominal pain	37 (90.2%)
Pericardial tamponade	6 (14.6%)
Pleural effusion	9 (22%)
Atrial fibrillation	7 (17.1%)
Lower extremity ischemia	2 (4.9%)
Renal failure requiring hemodialysis	1 (2.4%)
Transient cerebral ischemic attack	5 (12.2%)
Myocardial ischemia	6 (14.6%)
Chronic kidney disease	3 (7.3%)
Hypertension	33 (80.5%)
Diabetes mellitus	2 (4.9%)
Hyperlipidemia	9 (22%)
Smoking	16 (39%)
Pneumonia	9 (22%)
Alcoholism	9 (22%)
Aortic rupture	0
Cardiopulmonary resuscitation	0
Marfan syndrome	1 (2.4%)
**Primary tear site**	
Ascending aorta	30 (73.2%)
Transverse arch	5 (12.2%)
Proximal descending thoracic aorta	4 (9.8%)
Unknown	2 (4.9%)
**Aortic regurgitation**	
None	9 (22%)
Mild	8 (19.5%)
Moderate	5 (12.2%)
Severe	19 (46.3%)
**LV function**	
Good (EF≥60%)	33 (80.5%)
Medium (EF 30~60%)	8 (19.5%)
**Maximal ascending aortic diameter (cm)**	
Mean ± SD	5.1 ± 0.9
Median (range)	5.0 (3.4–7.2)
History of CBV event	3 (7.3%)
History of coronary artery disease	2 (4.9%)
Previous cardiac surgery	1 (2.4%)

*SD, Standard deviation; BMI, body Mass Index; CBV, cerebrovascular; LV, left ventricular; EF, ejection fraction; Categorical values are n (%)*.

### Surgical Procedure

**Figure 1:**
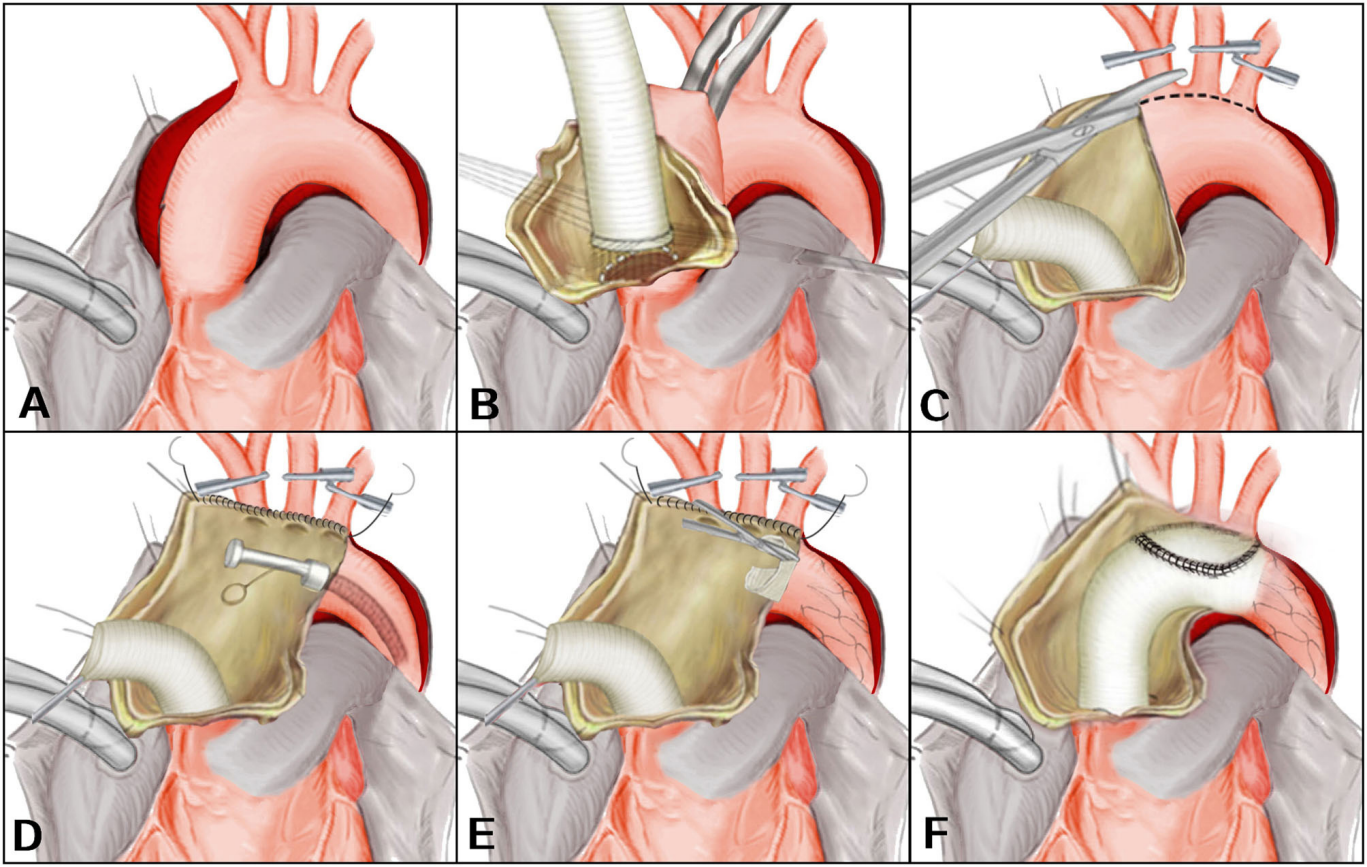
**(A–F)** En bloc reconstruction with the frozen elephant trunk technique. Revised from Jun-Ming Zhu, MD.

After the induction of general anesthesia, vascular cannulation in the left radial artery and dorsalis pedis artery was performed for continuous monitoring of arterial pressure. Cerebral perfusion was monitored throughout the procedure by near-infrared spectroscopy. Using conventional median sternotomy, the aortic arch and brachiocephalic vessels were dissected and exposed (Figure 1A). The cardiopulmonary bypass (CPB) was based on the right axillary artery and the femoral artery cannulation, and the right axillary artery was used for antegrade cerebral perfusion (ACP). Venous drainage was obtained with two-stage cannula in the right atrium. The left-heart venting catheter was inserted through the right superior pulmonary vein to prevent ventricular distension. When the patient was cooled to 32°C, the ascending aorta was clamped just proximal to the innominate artery. The proximal ascending aorta was longitudinally opened and cardioplegic solution was directly administrated via the coronary ostia for myocardia protection. The decision for reimplantation of the aortic valve, supra-commissural ascending aortic replacement or composite aortic root replacement was made after careful inspection of the morphological appearance of the cusps and root geometry (Figure 1B). During this period, the body temperature was continuously decreased. The brachiocephalic vessels were clamped when the proximal procedure was accomplished and the nasopharyngeal temperature reached 28°C. The circulatory arrest was instituted, and ACP was started at ~5 to 10 ml/kg per min.

After the ascending aorta was unclamped and opened, an incision was made on the anterior wall of the aortic arch longitudinally up to the origin of the descending aorta (Figure 1C). The arch vessels were excised as a unit from the superior surface of the dissected aortic arch and the separated layers of the aortic patch were reunited using continuous suture. A FET (Cronus, Microport Medical, Shanghai, China) was inserted anterogradely into the true lumen of proximal descending aorta. The proximal edge of the metallic stent (not the stent-free sewing edge) was located between the origin of the left subclavian artery and the descending aorta (Figure 1D). The stent-free sewing edge was pulled and trimmed to avoid blocking the origin of brachiocephalic arteries (Figure 1E). An opening corresponding to the size of the aortic patch surrounding the brachiocephalic arteries was made into the aortic graft which was anastomosed to the aortic root. The distal end of the aortic graft was anastomosed to the descending aorta containing the trimmed stent-free sewing edge. Then the aortic graft where an opening was made was anastomosed to the aortic patch (Figure 1F). CPB was gradually resumed to its normal flow, and rewarming was begun after the anastomosis was accomplished. A bovine pericardial patch and the remaining native aorta were sutured around the ascending aortic graft to control bleeding.

### Statistical Analysis and Follow-Up Methods

Continuous variables are expressed as the mean ± standard deviation and median (range), and categorical variables are presented as absolute values and percentages. Paired *t*-test were used to compare continuous variables. The Kaplan-Meier method was used to assess the cumulative survival rates. All statistical analyses were performed using SPSS 19 (SPSS Inc., Chicago, IL, USA). All statistical tests were 2-sided and *P*-value of <0.05 was considered statistically significant. Follow-up CTA was routinely performed before discharge, at 3, 6, and 12 months and annually thereafter.

## Results

From April 2018 to August 2020, 41 patients with acute Stanford type A dissection received total arch replacement using en bloc arch reconstruction and the FET technique at the first affiliated hospital of Zhengzhou University. Surgery time was 367–561 min (mean, 446 ± 46); CPB time was 172–335 min (means, 263 ± 44); aortic cross-clamp time was 87–213 min (means, 158 ± 38); and ACP time was 20–71 min (means, 44 ± 10). Concomitant procedures included the Bentall procedure in 8 patients, aortic valve reconstruction in 11 patients, supra-coronary aortic replacement in 22 patients, and coronary artery bypass graft in 1 patient. The operative data are summarized in Table 2.

**Table 2:** Surgical data.

**Variables**	**Mean ±SD (range)/*****n*** **(%)**
**Time of surgery (min)**	
Mean ± SD	446 ± 46
Median (range)	456 (367–561)
**Cardiopulmonary bypass time (min)**	
Mean ± SD	263 ± 44
Median (range)	276 (172–335)
**Aortic cross-clamp time (min)**	
Mean ± SD	158 ± 38
Median (range)	158 (87–213)
**Antegrade cerebral perfusion time (min)**	
Mean ± SD	44 ± 10
Median (range)	42 (20–71)
**Aortic root procedure**	
Bentall procedure	8 (19.5%)
Aortic valve reconstruction	11 (26.8%)
Supra-coronary aortic replacement	22 (53.7%)
Mitral valve repair	0
Coronary artery bypass graft	1 (2.4%)
**Diameter of FET**	
26 mm	31 (75.6%)
28 mm	10 (24.4%)
**Distal landing zone**	
T6	15 (36.6%)
T7	26 (63.4%)

*SD, Standard deviation*.

The postoperative hospital stay was 4 - 28 days, and the length of the intensive care unit stay was 1–8 days. The ventilation was required for a media 21 h after operation (range 7–243 h). In-hospital mortality was 9.8% (4 patients). The causes of death included low output syndrome (2 patients), multiple-organ failure (1 patient), and intractable hemorrhage (1 patient). Stroke was observed in 2 (4.9%) patients who recovered before hospital discharge. Paraplegia was observed in 1 (4.2%) patient and pneumonia was observed in 4 (9.8%) patients. Acute kidney injury (AKI) which was defined according to the RIFLE criteria was observed in 23 (56.1%) patients (9), and five patients presented with renal failure requiring hemodialysis. Two patients presented with tracheostomy and two patients required mediastinal re-exploration for hemorrhage and sternal dehiscence, respectively. Table 3 lists the major postoperative outcomes of this study.

**Table 3:** Postoperative outcomes.

**Event**	**Mean ± SD (range)/*****n*** **(%)**
**Length of ICU stay (days)**	
Mean ± SD	2.5 ± 1.7
Median (range)	2 (1–8)
**Ventilation (h)**	
Mean ± SD	37 ± 47
Median (range)	21 (7–243)
**Postoperative hospital stay (days)**	
Mean ± SD	14 ± 4.5
Median (range)	14 (4–28)
Pneumonia	4 (9.8%)
Acute kidney injury	23 (56.1%)
Renal failure requiring hemodialysis	5 (12.2%)
Tracheostomy	2 (4.9%)
Reoperation for bleeding	1 (2.4%)
Reoperation for sternal dehiscence	1 (2.4%)
Stroke	2 (4.9%)
Paraplegia	1 (2.4%)
In-hospital mortality	4 (9.8%)

*SD, Standard deviation; ICU, intensive care unit; Acute kidney injury was defined according to the RIFLE criteria*.

### Images

**Figure 2:**
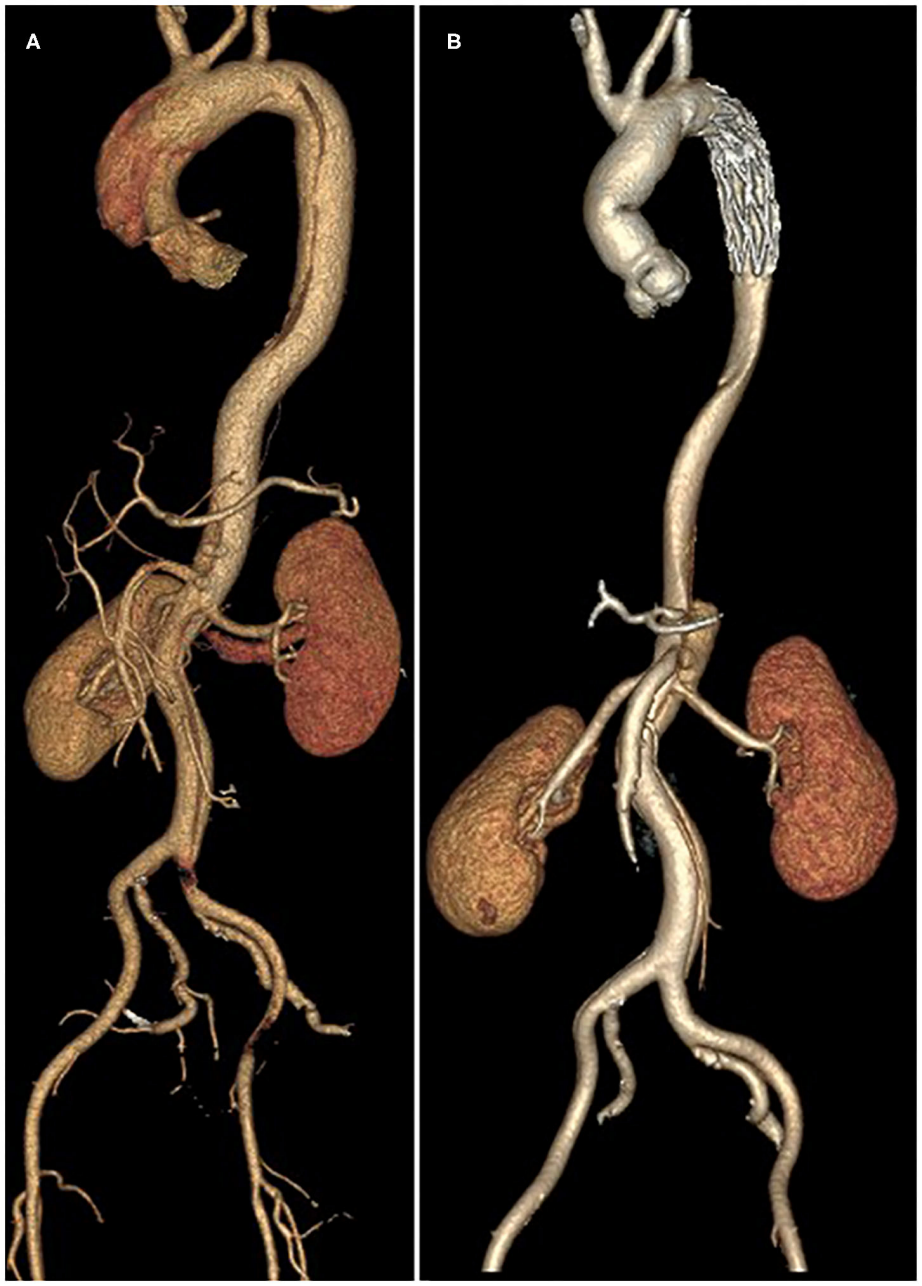
Preoperative **(A)** and postoperative **(B)** computed tomography angiography reconstruction.

**Figure 3:**
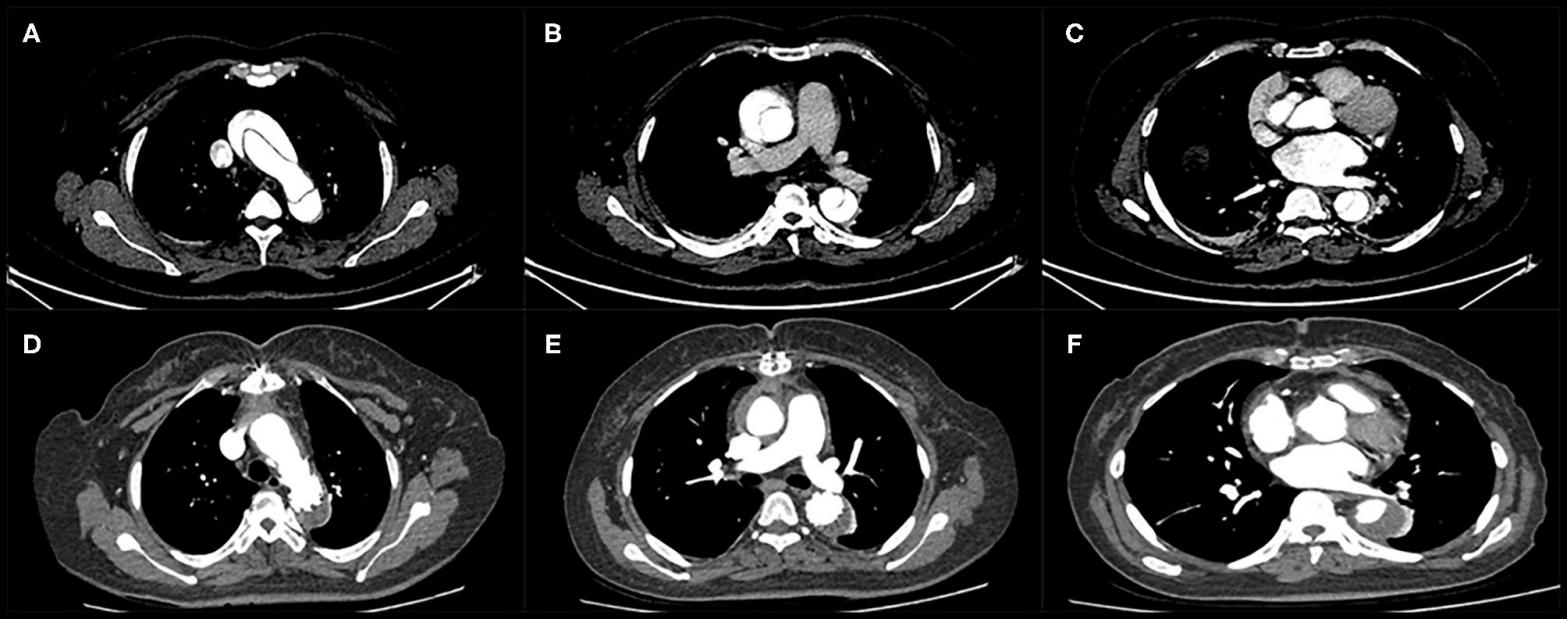
Computed tomography angiography of a patient with acute type A dissection before surgery **(A–C)** and during follow-up **(D–F)**. Thrombus formation in the false lumen was observed around the stented graft and distal to the edge of the stented graft.

Thirty-six patients underwent postoperative imaging with computed tomography which showed the enlargement of the true lumen, induction of thrombosis of the false lumen, and shrinkage of the aorta during follow-up. Preoperative and postoperative computed tomography angiography images are shown in Figures 2, 3. With regard to the final follow-up CTA, complete thrombus formation of the false lumen around the FET was observed in 91.6% of patients (33/36), at the diaphragmatic level in 69.4% of patients (25/36), and the superior mesenteric artery level in 27.8% of patients (10/36). The supra-aortic vessels were patent in 100% of patients (36/36) without stenosis. The true lumen diameter of the dissected aorta increased significantly from 10.7 ± 4.9 mm to 23.1 ± 8.9 mm at the pulmonary bifurcation level (*P* < 0.001), from 10.5 ± 4.4 mm to 17.5 ± 7.0 mm at the level of the diaphragm (*P* < 0.001), and from 9.6 ± 3.9 mm to 9.9 ± 4.0 mm at the superior mesenteric artery level (*P* < 0.001). The false lumen diameter of the dissected aorta decreased significantly from 14.8 ± 6.6 mm to 3.0 ± 1.7 mm at the pulmonary bifurcation level (*P* < 0.001), and from 12.4 ± 5.0 mm to 6.5 ± 3.2 mm at the diaphragm level (*P* < 0.001). At the superior mesenteric artery level the average diameter of false lumen did not significantly change from 10.7 ± 4.2 mm to 10.5 ± 4.1 mm (*P* = 0.06) (Table 4).

**Table 4:** Comparison preoperative and follow-up diameters (mm ± SD).

**Aortic level**	**Preoperative**	**Follow-up**	***P*** **-value**
**True lumen diameter**			
Pulmonary bifurcation	10.7 ± 4.9	23.1 ± 8.9	<0.001
Diaphragm	10.5 ± 4.4	17.5 ± 7.0	<0.001
Superior mesenteric artery	9.6 ± 3.9	9.9 ± 4.0	<0.001
**False lumen diameter**			
Pulmonary bifurcation	14.8 ± 6.6	3.0 ± 1.7	<0.001
Diaphragm	12.4 ± 5.0	6.5 ± 3.2	<0.001
Superior mesenteric artery	10.7 ± 4.2	10.5 ± 4.1	0.06

*SD, Standard deviation*.

### Follow-Up

**Figure 4:**
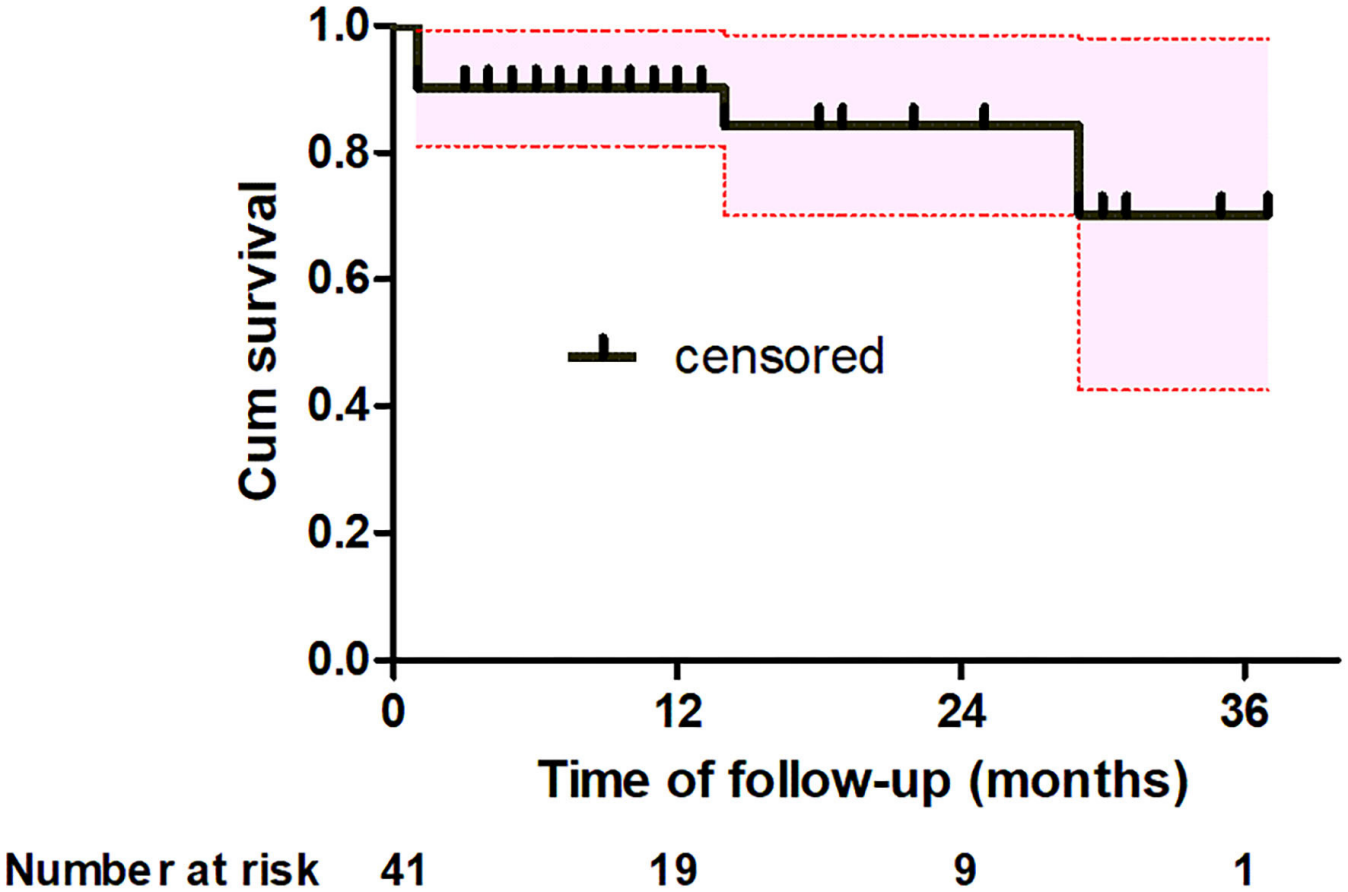
Kaplan-Meier analysis shows estimate of the actuarial survival (black solid line) for all patients with the 95% confidence interval (red dotted line).

One patient was lost during the follow-up. There were 2 late deaths during a mean of follow-up 13 ± 10 months (range, 1–37 months). One patient died of unknown causes 14 months after surgery, and the other patient died of lung cancer 29 months after surgery. Severe complications during follow-up were not observed. Kaplan–Meier analysis estimated that the actuarial survival rates at 1-, 2-and 3-year were 90.2% [95% confidence interval (CI), 81.2–99.2], 84.2% (95% CI, 70.1–98.3) and 70.2% (95% CI, 42.2–98), respectively (Figure 4).

## Discussion

In our present study, we reported our experience with the en bloc arch replacement combined with the FET technique in patients with acute type A dissection. Treatment of extensive diseased aorta involving the aortic arch and the descending aorta is a surgical challenge, and management of the dissected aortic arch is likely one of the most impelling. Some authors suggest only an ascending aortic replacement with or without proximal arch replacement to reduce postoperative mortality and morbidity rate (10), while other authors more aggressively support a total arch replacement by using of elephant trunk process to improve short- and long-term prognosis after operation (11, 12). The question of whether such an aggressive technique should be used in acute type A aortic dissection is still controversial. We described our technique for total aortic arch replacement: the main aortic graft was anastomosed to the supra-arch vessels in an island fashion. The technique needed to finish all the anastomoses before myocardial and lower body perfusion can be restored, namely proximal and distal anastomoses as well as an island reimplantation of the supra-aortic branches, so the CPB time and especially the myocardial ischemic time are potentially long. A disadvantage of this method may be leave behind the potentially diseased aortic wall on the area of the greater curvature. Patients with connective tissue disorders may be required for reoperation in the future because of the continued degeneration of the leaving native aortic tissue. There are only limited experiences with en bloc arch reconstruction combined with the FET technique in patients with connective tissue disorders in that there is only one patient with Marfan syndrome in this study. During the follow-up period, the evidence of progression of the aortic disease was not observed, but this does not give a clear indication on the prognosis of connective tissue disorders. In the meanwhile, to control the bleeding at the distal anastomosis or the posterior part of the island is technically demanding when the CPB has been resumed. For surgeon, bleeding from the anastomotic location maybe the most dreadful complication during aortic surgery due to the fragile aortic wall. The essential advantages of the en bloc arch reconstruction lie in only one anastomosis to be performed for the aortic arch vessels and long-term patency by virtue of the preservation of the native supra-aortic arteries. At present there are different methods for aortic arch reconstruction for acute type A aorticdissection, such as the *en bloc* (island) technique, the branched graft technique and the hybrid technique. Shrestha M and colleagues showed that the en bloc technique and the branched graft technique could achieve similar results for total aortic replacement (5), which was consistent with Schoenhoff et al. (13). Zhang et al. reported that compared with branched graft technique, hybrid aortic arch technique is a viable alternative treatment for patients with DeBakey type I aortic dissection (3). Li and his colleges compared the outcomes of the modified en bloc technique and the branched graft technique for acute type A aortic dissection and found that the modified en bloc technique was superior to the branched graft technique (7). In this series involving 41 patients undergoing island-technique for arch reconstruction with FET technique, the hospital mortality rate was 9.8%; Stroke and paraplegia rates were 4.9 and 2.4%, respectively. Re-thoracotomy due to bleeding occurred in 1 patient. The results can be considered satisfactory with regard to both mortality and morbidity in accord with other reports in view of the pathologic background of the disease and the complex surgical procedures (14, 15).

Nowadays, there is still no agreement on the optimal way to provide brain protection during acute type A aortic dissection surgery and it is more complex with the presence of possible arch vessels dissection and brain damage due to insufficient blood supply. Retrograde cerebral perfusion with oxygenated blood through the superior vena cava is less popular (16), the application of ACP has strongly increased at many centers worldwide in recent years (17). Sufficient cerebral perfusion is considered mandatory during circulatory arrest, and the circle of Willis was abnormal in about 40–68% of patients with diseases of the aortic arch (18, 19). However, sufficient collaterals could be achieved and there was no clinical impact of abnormalities of the circle of Willis (18, 19). Several reports have showed that ACP can maintain nearly normal cerebral metabolism, relieve cerebral edema, reduce intracranial pressure and metabolic acidosis in animal models (20, 21), and the use of ACP can provide a mortality benefit in patients with acute type A dissection (22). Even though the technical details of ACP are still discussed, the management of temperature protocol and safe time limits for cerebral perfusion are yet to be defined. A report from El-Sayed Ahmad et al. found that more than 60 min of selective ACP and moderate-to-mild systemic hypothermic circulatory arrest can safely be applied to patients with acute type A dissection (23). A meta-analysis revealed that using bilateral perfusion during aortic surgery resulted in superior operative outcomes compared to using unilateral perfusion if circulatory arrest was prolonged (24). Several studies have shown that ACP in combination with warmer circulatory arrest temperatures can be safely and decrease the incidence of permanent neurologic deficit and other poor clinical outcomes (15, 25). In the present series mean duration of ACP was 44 ± 10 min and core temperature was routinely cool to 28°C. The stroke rate was 4.9% and consistent with the stroke rate of 5% shown by De Bartolomeo et al. (2).

The ideal cannulation strategy in acute type A aortic dissection is another controversial topic, and each cannulation strategy has own advantages and drawbacks during CPB. In previous studies, axillary artery cannulation or femoral artery cannulation has been widely used and well-discussed (26–30). Axillary artery cannulation can avoid the disadvantages of retrograde perfusion, provide anterograde selective cerebral perfusion and achieved better neurological outcomes (26, 27, 30). The disadvantages of axillary artery cannulation are more time-consuming, greater technical demand and insufficient flow, which may affect the perfusion of organs (28). Femoral artery cannulation can be easily and safely performed. However, retrograde perfusion through the femoral artery might increase the risk for systemic malperfusion because of thrombus embolization and false lumen pressurization (29). Kreibich, M et al. reported because of quick and easy establishment of CPB central ascending aortic cannulation could be used as another option in patients with acute type A aortic dissection (31). Suenaga, E et al. found that 46 patients with acute type A aortic dissection underwent transapical aortic cannulation and gained acceptable early and mid-term outcomes (32). In our center, axillary artery and femoral artery cannulation were used to establish CPB in order to provide better systemic perfusion for acute type A aortic dissection repair. Huang et al. analyzed 327 patients who underwent surgical repair for type A aortic dissection using axillary artery in combination with femoral artery cannulation and showed relatively low incidences of early mortality (3.06%) and permanent neurologic dysfunction (0.92%) (33). The reason might be that the study consisted of different pathophysiological conditions, namely both acute and chronical cases. In a cohort of 476 patients with acute type A aortic dissection, the incidences of malperfusion-relation complications and in-hospital mortality were 18.1 and 13.5% in combined axillary artery and femoral artery cannulation group (34), which was similar to our study.

AKI is a common complication after aortic surgery which is more likely associated with high mortality. In present study, we observed that 23 patients presented AKI and five patients required continuous renal replacement therapy because of renal failure after operation, which is in accordance with the results reported by Li et al. (35). Open total aortic arch replacement is a complicated operation that includes the application of hypothermic circulatory arrest which can potentially lead to severe renal ischemic-reperfusion injury. Although the moderate hypothermic circulatory arrest has shortened the duration of CPB, the CPB duration is much longer compared with other cardiac surgeries and adds to the risk of AKI. Several studies had demonstrated that CPB duration was a predictor of AKI in patients undergoing cardiac and vascular surgery (36, 37). The potential mechanism was not clear, but one may speculate that CPB was associated with significant hemolysis which was related to the development of AKI after surgery. Another explanation is that cardiac surgery using CPB induces a systemic inflammatory response syndrome that may lead to tissue injury. Lannemyr et al. found that at the beginning of CPB renal tubular cell injury was detected with a peak biomarker increased early after the CPB in cardiac surgery (38). The renal ischemia—reperfusion injury may be the most important pathophysiologic processes which can cause acute renal failure. This means that the risk of renal tubular injury can be minimized by avoiding deep hypothermia and decreasing the CPB duration. Other risk factors of developing AKI underwent aortic arch surgery, which did have been found were older age, obesity, preoperative hypertension, chronic renal disease, emergency surgery, a higher number of red blood cell transfusion, and renal malperfusion due to the dissection itself or cardiac tamponade (35, 36, 39). As a result, emergency aortic surgery was strongly related to the development of postoperative AKI and might be known as a risk factor for AKI. In this series renal blood flow was restored after operation and one-fourth of our renal malperfusion patients did not develop postoperative AKI.

Postoperative patency and thrombosis of the false lumen is another important focus after acute type A aortic dissection repair. The FET technique is a useful way to seal the primary entry tear in the proximal descending aorta, reduce pressure in the false lumen to prevent its dilatation, and limit the risk of distal aortic reintervention (11, 12, 40). However, owing to continued false lumen perfusion through distal entry tears complete thrombosis of the false lumen does not always be achieved through the use of the FET technique. A meta-analysis indicated that compared with complete thrombosis, a residual patent lumen after repair of Stanford type A dissection is a significant independent predictor of long-term mortality and aortic events, and partial false lumen thrombosis is not associated with the long-term mortality (41). Increasing pressure in the false lumen can lead to the dilation of the aorta which increases wall tension and adds the risk of aneurysm expansion and rupture. Because of this, the second-stage repair may be needed after the primary operation. In our series, no patients received aortic reintervention during the follow-up period. The distal landing zone of the FET prosthesis was located at T6-T7 and paraplegia was observed in one patient in this study. Coverage of numerous potentially critical intercostal arteries by the stent graft is believed to pose an increased risk for paraplegia (42). Using FET the intercostal arteries below the T8 level could not be occluded in the present study. One potential explanation is that intercostal arteries arising from the thrombosis of false lumen and insufficient collateral circulation resulted in spinal cord ischemia. This paper describes our experience of applying the FET technique and the island process for arch vessel reimplantation for acute type A dissection. During the follow-up period complete false lumen thrombosis was observed in 91.6%, 69.4 and 27.8% of the patients at the pulmonary bifurcation level, the diaphragmatic level and the superior mesenteric artery level, respectively. The true lumen diameter significantly increased the false lumen diameter showed shrinkage at the same three levels of the descending aorta.

## Study Limitations

This study has several limitations: including small size sample, limited follow-up period, no comparative group and retrospective review of a single center’s experiences.

## Conclusions

The present retrospective study indicated that en bloc arch reconstruction with the FET procedure is feasible and effective for acute type A dissection. Early clinical follow-up results displayed that this procedure can be safely performed without reoperation. Further studies involving larger numbers of patients with a longer follow-up period are required.

## Data Availability Statement

The raw data supporting the conclusions of this article will be made available by the authors, without undue reservation.

## Ethics Statement

The studies involving human participants were reviewed and approved by the Ethics Committee of the First Affiliated Hospital of Zhengzhou University (2020-KY-0310-002). The need for individual patient consent was waived due to the retrospective nature of the analysis.

## Author Contributions

PL and BW conceived the research question, conceived and designed the analysis. CL, HX, GZ, and FS undertook data collection and conducted the study. HZ and XY drafted the manuscript. All authors reviewed the results, commented on the manuscript, and approved the final manuscript.

## Conflict of Interest

The authors declare that the research was conducted in the absence of any commercial or financial relationships that could be construed as a potential conflict of interest.

## Publisher's Note

All claims expressed in this article are solely those of the authors and do not necessarily represent those of their affiliated organizations, or those of the publisher, the editors and the reviewers. Any product that may be evaluated in this article, or claim that may be made by its manufacturer, is not guaranteed or endorsed by the publisher.

## Abbreviations

FET: frozen elephant trunk
CTA: computed tomography angiography
CPB: cardiopulmonary bypass
ACP: antegrade cerebral perfusion
AKI: acute kidney injury.
